# The hidden messengers: cancer associated fibroblasts—derived exosomal miRNAs as key regulators of cancer malignancy

**DOI:** 10.3389/fcell.2024.1378302

**Published:** 2024-04-17

**Authors:** Zixuan Gou, Jiannan Li, Jianming Liu, Na Yang

**Affiliations:** ^1^ Bethune First Clinical School of Medicine, The First Hospital of Jilin University, Changchun, China; ^2^ Department of General Surgery, The Second Hospital of Jilin University, Changchun, China; ^3^ Department of Otolaryngology Head and Neck Surgery, China-Japan Union Hospital of Jilin University, Changchun, China; ^4^ Department of Clinical Pharmacy, The First Hospital of Jilin University, Changchun, China

**Keywords:** cancer-associated fibroblasts-derived exosomal miRNA, cancer malignant characteristics, dual regulatory functions, diagnosis, prognosis, therapy

## Abstract

Cancer-associated fibroblasts (CAFs), a class of stromal cells in the tumor microenvironment (TME), play a key role in controlling cancer cell invasion and metastasis, immune evasion, angiogenesis, and resistance to chemotherapy. CAFs mediate their activities by secreting soluble chemicals, releasing exosomes, and altering the extracellular matrix (ECM). Exosomes contain various biomolecules, such as nucleic acids, lipids, and proteins. microRNA (miRNA), a 22–26 nucleotide non-coding RNA, can regulate the cellular transcription processes. Studies have shown that miRNA-loaded exosomes secreted by CAFs engage in various regulatory communication networks with other TME constituents. This study focused on the roles of CAF-derived exosomal miRNAs in generating cancer malignant characteristics, including immune modulation, tumor growth, migration and invasion, epithelial-mesenchymal transition (EMT), and treatment resistance. This study thoroughly examines miRNA’s dual regulatory roles in promoting and suppressing cancer. Thus, changes in the CAF-derived exosomal miRNAs can be used as biomarkers for the diagnosis and prognosis of patients, and their specificity can be used to develop newer therapies. This review also discusses the pressing problems that require immediate attention, aiming to inspire researchers to explore more novel avenues in this field.

## 1 Introduction

The International Agency for Research on Cancer predicted that the global incidence of cancer will rise by 75% by 2030, reaching 22.2 million new cases (Thun et al., 2010). According to a recent editorial published in the Journal of the American Medical Association, cancer is estimated to cost $25.2 trillion between 2020 and 2050, 0.55% of the world’s yearly gross domestic product (Lopes, 2023). Cancer is a complex disease involving alterations in the genome with multiple mutations, leading to uncontrolled cell proliferation and morphological changes (Graham and Sottoriva, 2017). Most malignancies present numerous malignant characteristics, including uncontrolled reproductive capacity, enhanced invasion and metastasis, increased angiogenesis, cell death resistance, immune surveillance evasion, metabolic reprogramming, and treatment resistance (Curtius et al., 2018; Wu et al., 2019). The extent of these behaviors is determined by the interactions between different components of the tumor microenvironment (TME) (Lee and Cheah, 2019).

TME consists of immune cells (such as T and B lymphocytes, natural killer (NK) cells, and tumor-associated macrophages) and stromal cells (fibroblasts, mesenchymal stromal cells, pericytes, and adipocytes), which are present in the extracellular matrix (ECM) (Malla et al., 2021; Ali et al., 2022; Tiwari et al., 2022). The importance of cancer-associated fibroblasts (CAFs), a crucial part of TME, in tumor control cannot be disregarded.

CAFs, a diverse group of interstitial cells, can be classified into various subtypes based on the differential expression of specific biomarkers, each having unique functions and roles (Zhang M. et al., 2022). Fibroblasts are usually quiescent in normal tissues but can be activated during tissue injury (Raju et al., 2022). One of the primary sources of CAFs is the presence of these activated fibroblasts that are seen near cancer cells. CAFs are a type of mesenchymal cells exhibiting high levels of elasticity, flexibility, and universality. They are actively engaged in cancer development by enhancing immune evasion, inducing angiogenesis, encouraging/suppressing chemotherapy resistance, and facilitating/inhibiting cancer cell invasion and metastasis through intricate interactions with other cell types in the TME (Chen and Song, 2019). One significant way CAFs operate is through exosomes (Yang et al., 2017; Pan et al., 2022a). For instance, they can mediate the proliferation and invasion of bladder cancer cells (Yan et al., 2020).

Almost every cell type in the human body can release exosomes, a subtype of extracellular vesicles (EVs) (Doyle and Wang, 2019). Exosomes, having an average diameter of 30–150 nm, were initially identified by Trams et al. In 1981 as cell-shedding vesicles that could be separated from various normal and malignant cells (Trams et al., 1981). These membrane-based vesicles were identified and given the formal term “exosomes” by Johnstone et al. in 1987 (Johnstone et al., 1987). Presently, exosomes are included into the small extracellular vesicles category according to MISEV guidelines and current consensus in the field. Exosomes contain proteins, lipids, and nucleic acids that can be transferred between cells. This transfer can activate various signaling pathways and regulate the biological functions of tumor cells. These exosomes have potential applications in tumor detection and management (Kalluri and LeBleu, 2020; Shao et al., 2020; Zhang et al., 2022b). In essence, the potential impacts of the contents of exosomes determine the manifestation of its regulatory influence.

The human body contains significant amounts of microRNA (miRNAs), vital in regulating the biological genome. Their expression profiles in cells vary between the normal and disease states in humans, suggesting that miRNAs can serve as disease biomarkers and have garnered growing interest (He et al., 2020; Hill and Tran, 2021; Liu J. et al., 2023). miRNAs significantly impact various essential biological processes, such as cell differentiation, apoptosis, proliferation, metabolism, and differentiation. They have also been linked to several illnesses, including malignancies (Iacona and Lutz, 2019; Li B. et al., 2021; Pan G. et al., 2023; Pan Z. et al., 2023). Exosomes provide an optimal environment for carrying miRNA because miRNA is unstable when it exists alone *in vitro* and can be degraded by RNA enzymes in the human body (Qiu et al., 2022). Several studies have pointed out that miRNAs in CAF-derived exosomes play an essential role in tumor regulation, and these miRNAs can interact with different cells in the TME through exosomes (Villegas-Pineda et al., 2021). The impact of extracellular miRNAs secreted by immune cells, cancer cells, and other cell types in TME has been briefly outlined by others and will not be further discussed in this work (Alizadeh et al., 2019; Xin et al., 2021; Hao et al., 2023). This review focuses on the function of CAF-derived exosomal miRNAs in cancer progression. Furthermore, it points out their prospects as tools for diagnosing and treating patients with cancer, in addition to acknowledging the pressing problems in the current research.

## 2 CAFs

CAFs are a type of stromal cells in the TME that can produce ECM related to cancer growth, invasion, and metastasis. Unlike normal fibroblasts, CAFs have been reprogrammed by the cancer cells and surrounding TME to promote tumor progression (Sahai et al., 2020). Activated fibroblasts, increased microvascular density, inflammatory cell count, and altered ECM composition are the salient characteristics of mesenchymal TME (Huang et al., 2021; Bilotta et al., 2022; Jayaram and Phillips, 2024). Since CAFs are a form of interstitial cells with high universality, plasticity, and elasticity, they actively participate in cancer development through intricate interactions with other cell types in the TME (Mao et al., 2021). The characteristics and interactions of CAFs with different cell types may undergo dynamic changes as tumor participants. Fibroblast activating protein (FAP) and α-smooth muscle actin (α-SMA) expression are commonly used to identify CAFs (Geng et al., 2021). CAF’s effect on tumors is currently under extensive investigation, as it plays a crucial role in the development and advancement of cancer. Consequently, it is considered a highly promising target for cancer treatment.

### 2.1 Sources of CAFs

Generally, fibroblasts are not endothelial, immune, or epithelial cells but mesenchymal cells (Wei et al., 2021). Hence, fibroblasts present inside or close to the tumor can be considered CAFs (Papait et al., 2022). However, the exact cellular origin and function of fibroblasts remain unclear and difficult to determine because of their phenotypic and functional heterogeneity.

**FIGURE 1:**
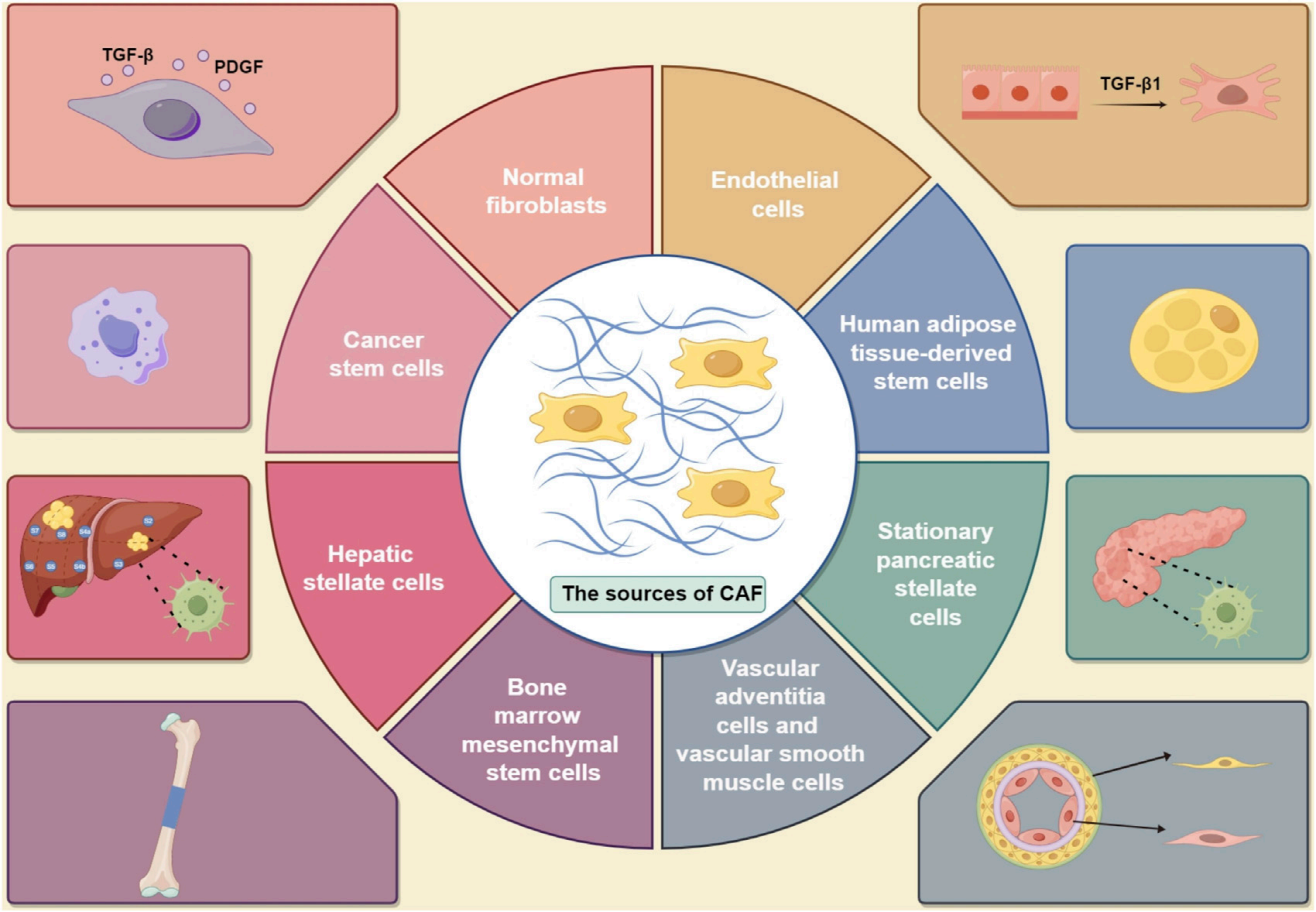
Sources of CAFs in tumors, including (1) normal fibroblasts, which can be influenced by TGF-β and PDGF (O’Connor et al., 2023; Buechler and Turley, 2018); (2) cancer stem cells (Liubomirski et al., 2019); (3) vascular adventitia and vascular smooth muscle cells (Zeltz et al., 2019); (4) endothelial cells, which can be activated by TGF- β1 (Chu et al., 2022; Shinkawa et al., 2022); (5) human adipose tissue-derived stem cells (Sato et al., 2023); (6) stationary pancreatic stellate cells (Morgan et al., 2023); (7) hepatic stellate cells (Wang S. S. et al., 2021); and (8) bone marrow mesenchymal stem cells (TGF- β: transforming growth factor-β; TGF- β1: transforming growth factor-β1; PDGF: platelet-derived growth factor). (By Figdraw).

According to the findings of published studies, the primary sources of CAFs are as follows: (1) resident fibroblasts (O’Connor et al., 2023; Buechler and Turley, 2018); (2) bone marrow mesenchymal stem cells (Liubomirski et al., 2019); (3) vascular adventitia and smooth muscle cells (Zeltz et al., 2019); (4) endothelial cells (Chu et al., 2022; Shinkawa et al., 2022); (5) human adipose tissue-derived stem cells (Sato et al., 2023); (6) stationary pancreatic stellate cells (Morgan et al., 2023) and hepatic stellate cells (Yin et al., 2013; Wang S. S. et al., 2021; Sankar et al., 2023); and (7) cancer stem cells (Najafi et al., 2019) (Figure 1). Understanding the origins of different CAFs can shed light on their functions and phenotypes. This will contribute to advancing research on targeted tumor therapies involving CAF-derived cells.

### 2.2 Heterogeneity of CAFs

Studies have indicated that heterogeneity in CAFs primarily manifests through variations in cellular phenotype. The phenotypic changes in CAFs exhibit temporal and geographical features, which involve the development of distinct fibroblast phenotypes and the differentiation of phenotypes of the same fibroblast in different tissue regions (Sahai et al., 2020; Zhang et al., 2022c). The advancement in single-cell RNA sequencing technology has enabled the statistical evaluation of transcriptome variations at the cellular level (Li X. et al., 2022; Luo et al., 2022). This analysis technique reveals the coexistence of various fibroblast populations in CAFs, forming different subgroups (Mezawa and Orimo, 2022). The main subgroups of CAFs include (1) mCAF, derived from resident tissue fibroblasts, mainly present in the periphery of tumors, involved in immune suppression (Zhang Z. et al., 2020); (2) dCAF, originating from tumor epithelial stroma, located close to cancer cells in the early stages of tumors, promoting tumor cell migration (Ohlund et al., 2017); (3) vCAF, converted from perivascular cells. In the early stages of tumor development, vCAFs are located near blood vessels, but during tumor evolution, they are located within the stromal compartment, promoting angiogenesis (Bartoschek et al., 2018; Mezawa and Orimo, 2022); (4) cCAF, overlapping with vCAF and exhibiting strong proliferative ability (Wang et al., 2022a); (5) iCAF, associated with inflammation, characterized by immune regulatory molecules, involved in tumor metastasis, immune suppression, migration, and other processes (Chen Z. et al., 2020); (6) myCAF, derived from myofibroblasts (Ohlund et al., 2017; Peltier et al., 2022), involved in tumor cell metastasis and drug resistance (Das et al., 2020); (7) apCAF, the antigen-presenting CAF population, capable of presenting antigens to T cells. However, due to the lack of necessary co-stimulatory molecules such as CD80, CD86, and CD40, they can hardly induce T cell clonal proliferation. Existing studies hypothesize that apCAFs act as decoy receptors, inhibiting immune reactions in the tumor microenvironment (Elyada et al., 2019). Furthermore, other CAF subtypes, including CD146+ CAFs (Brechbuhl et al., 2017), α-SMA+ CAFs (McAndrews et al., 2022), asporin+ CAFs (Maris et al., 2015), and versican+ CAFs (Fanhchaksai et al., 2016), also play tumor-suppressive roles. In summary, there are significant variations in the overall survival rates, immune cell counts, and response rates to immunotherapy among different subtypes (Wang et al., 2022a).

Markers used to identify different subtypes of CAF are: (1) surface markers such as podoplanin (PDPN), fibroblast activating protein α (FAP-α), platelet-derived growth factor receptor α or β (PDGFR α or β), TGF-β receptor I/II (TGF-βR I/II), epidermal growth factor receptor (EGFR), fibroblast growth factor receptor (FGFR), and bone morphogenetic protein receptor I/II (BMPR I/II); (2) intracellular markers such as α-SMA, actin-α cardiac muscle (ACTA2), S100A4, fibroblast specific protein-1 (FSP-1), vimentin (VIM), desmin, and transgelin (TAGLN); (3) extracellular biomarkers such as collagen 1a1 (COL1A1), COL1A2, lumican (LUM), decorin (DCN), microfibril associated protein 5 (MFAP5), fibronectin, tenacin-C, periostin, and remodeling enzymes: lysyl oxidase (LOX), lysyl oxidase-like 1 (LOXL 1), and matrix metalloproteinase (MMP); and (4) growth factors and cytokines: TGF-β, vascular endothelial growth factor (VEGF), platelet-derived growth factor (PDGF), fibroblast growth factor (FGF), paternally expressed gene 2 (PEG2), connective tissue growth factor (CTGF), stromal cell-derived factor-1 (SDF-1), and WNT, and so on (Dzobo and Dandara, 2020; Aden et al., 2023; Xu et al., 2023).

Additional research stemming from the diversity of CAFs includes whether the development of cancer or mutations in tumor suppressor genes within tumor cells can lead to the transformation of one subtype into another or the emergence of new subtypes. Furthermore, the precise mechanisms and interactions through which different subtypes of CAFs regulate tumors are currently being investigated.

### 2.3 Functions of CAFs in TME

TME is a complex network of cells embedded in the ECM, playing a crucial role in tumor formation. These include immunological and endothelial cells, adipocytes, tumor cells, and CAFs. The presence of activated fibroblasts, higher density of microvascularization, inflammatory cell count, and altered ECM components are the key characteristics of TME (Rimal et al., 2022). Of all the mesenchymal cells that make up TME, CAFs are the most common cells (Fang et al., 2023). The contributions of CAFs vary according to tumor type. For example, pancreatic adenocarcinoma displays a highly reactive stroma where CAFs are thought to play a prominent role in tumor progression. But, in other tumor types, their presence is quite restricted (Geng et al., 2021; Aden et al., 2023). So far, their role in tumor development has not been fully understood.

CAFs have higher contractility, increased proliferation rate, and greater expression of α-SMA than normal fibroblasts, suggesting its undeniable effect on tumor genesis and development (Affo et al., 2017). According to recent studies, CAFs can produce ECM in the TME that sustains tumors, encourages pre-tumor epithelial cell growth, expansion, and diffusion, fosters the emergence of newly malignant cells, and significantly impacts the progression of different organ tumors (Wang Y. et al., 2022; Sun et al., 2022). Through indirect cellular contact, CAFs can also control the biological activity of other mesenchymal and tumor cells, reshape and synthesize ECM, and release many regulatory factors, which can affect the development and progression of tumors (Zhang M. et al., 2022; Wu et al., 2022). In addition, it can reprogram lipid metabolism, release lipid metabolites, get absorbed into tumor cells, encourage migration, and so forth (Gong et al., 2020). Other mechanisms include immune regulation and extracellular secretion (Peng et al., 2023). CAFs have been shown to play an immunosuppressive role in the TME through the secretion of several key regulatory factors, including cytokines and chemokines. Cytokines involved include TGF-β, IL-6, IL-33, TGF-β-induced gene (TGFBI), cardiotrophin-like cytokine factor 1 (CLCF1), TNF, and IL-1β (Limoge et al., 2017; Karakasheva et al., 2018; Shani et al., 2020; Sato et al., 2021; Xiang et al., 2022). Chemokines such as CXCL12, CCL2, CCL17, and CXCL16 are also involved (Chun et al., 2015; Xiang et al., 2022; Zheng and Hao, 2023). The following text will elaborate on how exosomes secrete and govern malignancies.

Recently, researchers have become interested in CAFs that exhibit an aging phenotype. Under stress conditions, these cells can exhibit a senescence-associated secretory phenotype (SASP), leading to cancer progression and chemotherapy resistance. An interesting study in a mouse model found that CAFs with SASP can promote the formation of peritoneal tumors through the JAK/STAT3 signaling pathway. Furthermore, the presence of CAFs with SASP was discovered in the ascitic fluid of gastric cancer (GC) patients with peritoneal metastasis. The results suggest that CAFs with SASP may promote peritoneal dissemination of primary tumors and cancer progression (Yasuda et al., 2021). Another study demonstrated that the proliferative potential of pancreatic cancer (PC) may be associated with p53-mediated cellular senescence and CAFs with SASP (Higashiguchi et al., 2023). However, the role of aged CAFs in metastatic lesions and the molecular mechanisms induced by inflammation-related SASP still require further investigation.

In general, CAFs impact tumor formation in TME through invasion and metastasis promotion, angiogenesis, stem cell properties of cancer cells, and resistance to chemotherapy and radiation therapy. However, the unique internal mechanics of CAFs are not fully understood.

## 3 Exosome

### 3.1 Structure of exosome

In recent years, exosomes have garnered increasing attention in tumor research due to their role in transporting different active compounds from cells, influencing immune escape, tumorigenesis, and TME reconstruction (Dzobo and Dandara, 2020). Other types of cells can secrete exosomes, which are extracellular vesicles with a diameter ranging from 30 to 150 nm. They can be found in blood, breast milk, urine, nasal secretions, pleural effusion, bronchoalveolar lavage, and ascites, among other body fluids. Exosomes have a highly stable structure as their membrane lipid bilayers have rich ceramide, sphingomyelin, cholesterol content, and numerous transmembrane proteins. Exosomes contain proteins, lipids, DNA, and RNA (mRNA, miRNA, and other non-coding RNAs), which are crucial to the biological functions of exosomes (Affo et al., 2017; Rimal et al., 2022; Fang et al., 2023). Exosomes originate from within cells, and their biological processes primarily involve two main pathways: the endosomal and multivesicular body pathways (Arya et al., 2024). They mainly regulate receptor cells by two mechanisms. First, they interact with the target cell receptors and trigger associated signaling pathways. Second, their contents are released following endocytosis or fusion with the target cell’s plasma membrane, resulting in modifications to the receptor cell’s protein translation and related gene expression levels (Zhu et al., 2020; Rajabi et al., 2022).

### 3.2 Role of exosomes in tumor progression

Exosomes take part in growth and carcinogenesis through multiple processes. (1) They can enhance cellular communication with target cells by directly interacting with extracellular receptors to transmit signals or fusing with cell membranes to absorb or internalize them (Gurung et al., 2021). (2) Substantial evidence suggests that exosomes produced by tumors can accelerate tumor cell growth and alter the migration direction of recipient malignant cells. (3) Through metastatic treatment resistance, exosomes can guarantee communication between tumors (Mashouri et al., 2019). (4) In controlled conditions, the exosomes secreted by epithelial cells can act as messengers, delivering inflammatory signals to immune cells throughout the body (Yang et al., 2021). (5) Exosomes can regulate the immune system through molecular transport and signal transduction (Xie et al., 2022). They can also regulate immune cell activity, which in turn stimulates the immune system to release tumor cells (Taha et al., 2019; Daassi et al., 2020). (6) They can induce fibroblasts to express matrix metalloproteinases, facilitate matrix remodeling, and eventually encourage tumor invasion (Bai et al., 2023). (7) Exosomes may also have a role in forming CAFs (Wang et al., 2018). (8) Exosomes can also exert tumor suppressor effects due to the differences in their contents (Yang Z. et al., 2020).

Currently, studies examining the connection between exosomes and cancers are delving further to improve tumor diagnosis and treatment. Tumor and immune cells can create special exosomes that can be used directly in anti-tumor immunotherapy (Zhang et al., 2023). In the future, exosomes may also be best utilized as a cancer vaccine (He et al., 2018; Huda and Nurunnabi, 2022). They are also beneficial diagnostic markers, offering a new technique for early tumor diagnosis (Yang D. et al., 2020).

## 4 miRNA

### 4.1 Structure and production mechanism of miRNA

Since they have a greater prevalence in body tissues and fluids, significant impact on gene expression, and potential applications as disease biomarkers, miRNAs have become a fascinating topic for basic and translational biomedical research (Gjorgjieva et al., 2019). miRNA, a non-coding RNA of 22–26 nucleotides, constitutes 1% of the human genome’s total number of genes. By binding to the target gene’s untranslated 3′UTR region, it inhibits the target gene’s transcription, modifying gene expression levels and ultimately influencing intracellular homeostasis, a method by which eukaryotic cells control the gene transcription (Fuhrmann and Brune, 2022). miRNAs regulate many essential biological functions, including differentiation, apoptosis, proliferation, and metabolism. Several miRNAs have the potential to cause cancer and are strongly linked to tumor development, making them valuable as diagnostic markers and therapeutic targets (Breulmann et al., 2023). Primary miRNA, or pri-miRNA, is created when RNA polymerase II (RNA pol II) transcribes the miRNA genes inside and between genes. Drosha-DGCR8 then processes the pri-miRNA to pre-miRNA in the nucleus, which is then transported to the cytoplasm by Exportin5/RanGTP. This is the current understanding of the mechanism underlying the production and function of miRNAs. Dicer, a naturally occurring ribonuclease in the cytoplasm, further cleaves and converts the pre-miRNAs into mature, short, double-stranded RNA fragments (∼22 nt) called miRNAs. To exert its regulatory function, one strand of the mature miRNA duplex binds to the argonaute protein (AGO2) in RNA-induced silencing complex (RISC) and a complementary site in the 3′UTR of the target mRNA, resulting in translation inhibition. The second strand is degraded, leading to mRNA cleavage and generation of cytoplasmic processing bodies, known as P bodies (sites of mRNA decay and inhibition) (Iqbal et al., 2019; Long et al., 2023; Rezaee et al., 2023).

miRNA can exist in body fluids in various forms, and exosomes are one of the common packaging ways (Cortez et al., 2011). The sorting and packaging of miRNAs in exosomes is essential in comprehending vesicles’ role in cancer. Research has shown that stress-induced microenvironment can trigger miRNA trafficking and packaging into exosomes (Lee et al., 2017). Furthermore, evidence suggests that RNA-binding proteins play a synergistic role in the biogenesis of exosomes containing miRNAs. These include heterogeneous nuclear ribonucleoprotein-A2B1 (hnRNPA2B1) (Groot and Lee, 2020), MEX3C RNA binding protein (Lu et al., 2017), Y-box binding proteins (Shurtleff et al., 2016), Argonaute-2 (McKenzie et al., 2016), and membranous proteins such as caveolin-1 (Giannubilo et al., 2024).

### 4.2 Role of miRNA in tumors

The miRNAs expressed in cancer cells can participate in tumor progression through dual effects of carcinogenesis or tumor suppression (Hill and Tran, 2021). These processes can be achieved through epigenetic modifications, such as widespread genomic DNA hypomethylation (Ma et al., 2023) and histone acetylation (Liu Y. et al., 2022). In addition, transcription factors c-myc and p53 can also participate in tumor regulation through miRNA interactions (Parfenyev et al., 2021; Li Z. Y. et al., 2022; Kaller et al., 2022). The specific roles of miRNAs in tumors mainly include migration and invasion, tumor cell proliferation, and drug resistance. For example, miR-144-3p can induce iron deficiency by negatively regulating the expression of ZEB1, thereby inhibiting the proliferation, migration, and invasion of osteosarcoma (OS) cells (Jiang M. et al., 2023). miR-874-3p can participate in the migration, invasion, and proliferation of breast cancer cells by targeting voltage-dependent anion channel 1 (VDAC1) (Yang et al., 2023). miR-223-3p regulates ECT2 to promote GC proliferation, invasion, and metastasis through the Wnt/β-catenin signaling pathway (Li et al., 2023). Hence, it is evident that miRNAs are closely related to tumor development.

A large number of studies show that the expression profiles of miRNAs in cells under human disease conditions differ from those in normal conditions, indicating that miRNAs have the potential to become markers for disease diagnosis and provide a theoretical basis for disease liquid biopsy (Moro et al., 2023).

## 5 Function of CAF-derived exosomal miRNA in promoting tumorigenesis and development

### 5.1 Role in EMT

EMT was initially conceptualized as how tumor cells changed from an epithelial to a mesenchymal phenotype. According to recent studies, it also involves a continuous process known as partial EMT or intermediate mixed epithelial and mesenchymal (E/M) phenotypes (Taki et al., 2021). The process results in a loss of epithelial integrity and characteristics, the acquisition of mesenchymal properties, reduction in intercellular connections, decreased interactions with surrounding and stromal cells, and increased cell motility and migration (Dongre and Weinberg, 2019). Cancer overtakes this process to induce fundamental alterations in cell motility and shape, thereby facilitating invasion (Lu and Kang, 2019). Furthermore, research suggests that EMT orchestrates various complementary characteristics of cancer, including stem cell properties, tumorigenicity, metabolic reprogramming, treatment resistance, and the ability of tumor cells to adapt to changes in their microenvironment (Jiang et al., 2021; Qin et al., 2021; Manfioletti and Fedele, 2022). Numerous studies now demonstrate the involvement of miRNAs, along with factors such as vascular endothelial growth factor, WNT, Notch, PDGF, and epidermal growth factor, as well as external factors like alcohol and UV light, and pathological conditions including hypoxia in the TME, in the activation mechanism of the EMT process (Behbahani et al., 2017; Sugita et al., 2022; Yang et al., 2022).

**FIGURE 2:**
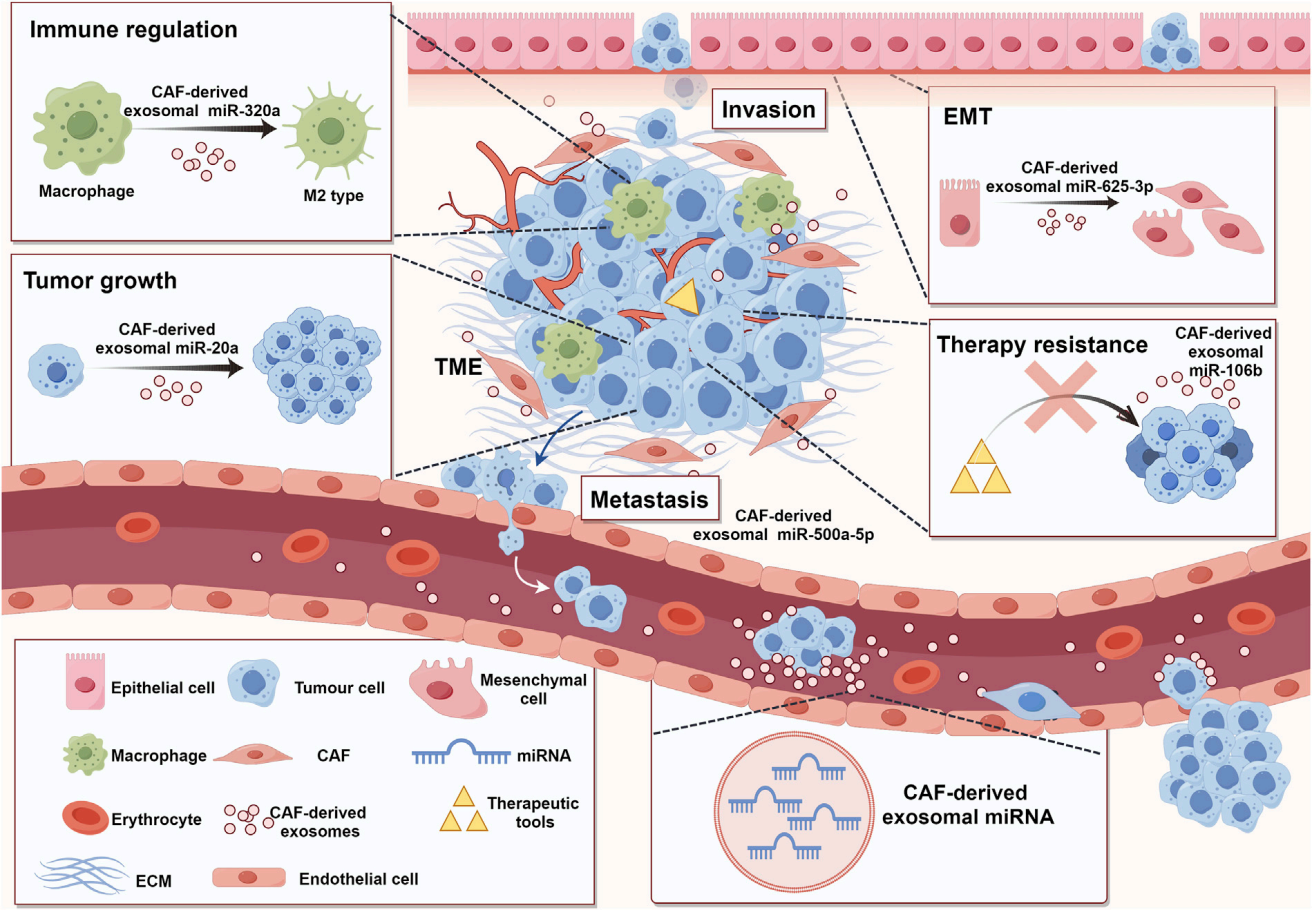
Functions of CAF-derived exosomal miRNA in promoting tumorigenesis and cancer development. TME comprises various cell types, including stromal cells, immune cells, tumor cells, and so on. All these cells were enveloped in ECM (Fang et al., 2023). CAFs, as an important component of TME, can participate in tumor regulation by secreting exosomal miRNAs (Wang J. W. et al., 2019). These miRNAs can take part in immune modulation (Zhao et al., 2022), tumor growth (Shi L. et al., 2022), migration and invasion (Chen B. et al., 2021), EMT (Zhang Y. et al., 2022), and treatment resistance (Fang et al., 2019) (ECM: extracellular matrix; TME: tumor microenvironment; EMT: epithelial-mesenchymal transition). (By Figdraw).

Exosomal miRNAs generated from CAFs also play a role in this process. Globally, the second most prevalent cause of cancer-related mortality is colorectal cancer (CRC). Studies have demonstrated that exogenous miR-625-3p produced by CAFs may stimulate EMT in CRC cells by blocking the CELF2/WWOX pathway (Zhang Y. et al., 2022). Similarly, in CRC, exosomes produced from CAFs express more miR-92a-3p when the Wnt/β-catenin pathway is activated. Thus, this leads to EMT in CRC cells, inhibiting mitochondrial apoptosis and directly suppressing FBXW7 and MOAP1 (Hu et al., 2019), providing potential candidates for CRC prediction and treatment. Breast cancer tissue has low expression of tumor suppressor HOXA5. *In vitro*, its overexpression causes cancer cells to undergo EMT inhibition and accelerate apoptosis (Hussain et al., 2020; Lu et al., 2021). A study involving 122 patients with surgically removed cancerous tissues and corresponding paracancerous tissues revealed that miR-181d-5p in exosomes derived from CAFs can target transcription factors that bind to the HOXA5 promoter, thereby stimulating the growth of MCF-7 cells and inhibiting their apoptosis, thus playing a pivotal role in the environmental effects of tumors (Wang et al., 2020). Similarly, the exosomal miR-18b selectively binds to the transcription elongation factor TCEAL7’s 3′UTR region, activating NF-κB. It encourages nuclear Snails to become ectopically active, causing EMT in breast cancer cells (Yan et al., 2021). Furthermore, Josson et al. used laser capture anatomical microscopy to isolate the cancer-related prostate stromal fibroblasts and bone-related stromal models. The exosome-derived miR-409-3p and miR-409-5p specifically upregulated delta-like one homologous deiodinase, iodothyronine 3 (DLK1-DIO3), which was involved in the regulation of developmental and embryonic processes on human chromosome 14. These expressions inhibited genes suppressing tumor growth, such as Ras, promoting tumor induction and EMT *in vitro* and *in vivo* (Josson et al., 2015). Some studies have also reported on the EMT of tumors, as shown in Table 1 (Li et al., 2019) (Figure 2).

**TABLE 1:** Summary of CAF-derived exosomal miRNAs’ functions in the malignant characteristics of a tumor.

Malignant characteristics	Tumor type	miRNA	Expression	Mechanism	References
EMT	Colorectal cancer	miR-625-3p	Upregulated	Blocks the CELF2/WWOX pathway	Zhang et al. (2022d)
		miR-92a-3p	Upregulated	Inhibits FBXW7 and MOAP1 and activates Wnt/β-catenin pathway	Hu et al. (2019)
	Breast cancer	miR-181d-5p	Upregulated	Targets transcription factors that bind to the HOXA5 promoter, thereby stimulating the growth of cancer cells and inhibiting their apoptosis	Wang et al. (2020)
		miR-18b	Upregulated	Activates NF-κB and encourages nuclear Snail to become ectopically activated	Yan et al. (2021)
	Prostate cancer	miR-409-3p	Upregulated	Inhibit Ras suppressor 1 and stromal antigen 2	Josson et al. (2015)
		miR-409-5p			
	Endometrial cancer	miR-148b	Downregulated	Binds to its downstream target DNMT1	Li et al. (2019)
Invasion and migration	Clear cell renal cell carcinoma	miR-224-5p	Upregulated	Internalizes itself to take part in controlling the cell invasion and migration	Liu et al. (2021)
	Esophageal cancer	miR-3656	Upregulated	Downregulates ACAP2 to improve the activation of the β-catenin and PI3K/AKT signaling pathways	Jin et al. (2020a)
	Pancreatic cancer	miR-421	Upregulated	Promotes glycolysis by regulating the SIRT3/H3K9Ac/HIF-1α axis	Zhou et al. (2022)
	Non-small cell lung cancer	miR-210	Upregulated	Inducts the PTEN/PI3K/AKT pathway	Yang et al. (2020c)
	Gastric cancer	miR-29b-1-5p	Upregulated	Mimics tumor angiogenesis and suppresses cell death via the 1/zonal occluden-1 axis	Wu et al. (2023)
	Osteosarcoma	miR-1228	Upregulated	Downregulates endogenous SCAI mRNA and protein levels	Wang et al. (2019a)
	Colorectal cancer	miR-345-5p	Upregulated	Interacts with CDKN1A to promote CRC progression and metastasis	Shi et al. (2023)
		miR-21	Upregulated	Directly increases the proliferative and invasive capacity of the cells	Bhome et al. (2017)
		miR-17-5p	Upregulated	Targets the 3′UTRs of RUNX3 by activating TGF-β signal pathways, and autocrine TGF-β activates CAFs through the RUNX3/MYC/TGF-β1 signal	Zhang et al. (2020b)
	Oral squamous cell carcinoma	miR-382-5p	Upregulated	Targets PTEN, YBX1, RUNX1, STC1, JAM2, and MMP16 promoting migration and invasion	Sun et al. (2019)
		miR-146b-5p	Upregulated	Suppresses HIPK3	He et al. (2023)
	Liver cancer	miR-329-3p	Upregulated	Inhibit the expression of HHIP, weaken cell adhesion	Jin et al. (2022)
		miR-380-3p			
		miR-410-5p			
		miR-431-5p			
		miR-20a-5	Upregulated	Targets LIMA1 to inhibit the Wnt/β-catenin signaling pathway	Qi et al. (2022)
	Lung squamous cell carcinoma	miR-369	Upregulated	Acts via NF1-mediated MAPK signaling pathway	Guo et al. (2020)
	Prostate cancer	miR-146a-5p	Downregulated	Activates the EGFR/ERK pathway to prevent cells from metastasizing when subjected to ADT	Zhang et al. (2020d)
	Oral squamous cell cancer	miR-34a-5p	Downregulated	Binds to AXL and enhances β-catenin nuclear translocation, leading to the overexpression of *SNAIL* transcription and the subsequent activation of MMP-2 and MMP-9	Li et al. (2018)
	Ovarian cancer	miR-29c-3p	Downregulated	Activates matrix metalloproteinase 2	Han et al. (2023)
	Breast cancer	miR-16	Downregulated	Suppress fibroblast-specific inducible focal adhesion kinase	Wu et al. (2020)
		miR-148a			
		miR-1-3p	Downregulated	Inhibits GLIS1	Tao et al. (2021)
	Triple-negative breast cancer	miR-4516	Downregulated	Prompts FOSL1’s tumor-promoting activity	Kim et al. (2020)
	Gastric cancer	miR-139	Downregulated	Lower the expression of MMP11 in the TME	Shi et al. (2020)
		miR-34			Xu et al. (2019)
Tumor angiogenesis	Multiple myeloma	miR-21	Upregulated	Increases the expression of alpha-smooth muscle actin and fibroblast activation protein	Miaomiao et al. (2023)
	Colorectal cancer	miR-135b-5p	Upregulated	Inhibits thioredoxin interacting proteins, downregulates FOXO1, and encourages the migration and proliferation of human umbilical vein endothelial cells	Yin et al. (2021)
					Dai et al. (2022)
Lymphatic metastasis	Esophageal cancer	miR-100-5p	Downregulated	Causes high expression of IGF1R/PI3K/AKT	Chen et al. (2023)
Tumorigenesis	Colorectal cancer	miR-200b-3p	Downregulated	Upregulates ZEB1 and E2F3	Yuan et al. (2022)
		miR-181b-3p	Upregulated	Controls the expression of SNX2	Jiang et al. (2023b)
Cell proliferation	Non-small cell lung cancer	miR-20a	Upregulated	Targets PTEN to increase the PI3K/AKT pathway’s activity	Shi et al. (2022b)
	Breast cancer	miR-500a-5p	Upregulated	Attaches to USP28, which promotes cell division	Chen et al. (2021a)
	Colorectal cancer	miR-135b-5p	Upregulated	Inhibits thioredoxin-interacting protein	Yin et al. (2021)
	Head and neck cancer	miR-3188	Downregulated	Directly targets B-cell lymphoma 2	Wang et al. (2019b)
	Intrahepatic cholangiocarcinoma	miR-195	Downregulated	Not clear	Li et al. (2017)
	Endometrial cancer	miR-320a	Downregulated	Inhibits the HIF1 α/VEGFA axis	Zhang et al. (2020e)
Metabolic alterations	Prostate cancer	miR-22	Upregulated	Decrease mitochondrial oxidative phosphorylation and cause changes akin to hypoxia in the TME	Zhao et al. (2016)
		miR-125b			
Generation of stem cell-like characteristics	Laryngeal cancer	miR-34c-5p	Upregulated	Unclear	Wang et al. (2022c)
	Colorectal cancer	miR-92a-3p	Upregulated	Suppresses FBXW7 and MOAP1 to prevent mitochondrial apoptosis	Hu et al. (2019)
Chemotherapy resistance	Pancreatic cancer	miR-106b	Upregulated	Targets TP53INP1 resulting in gemcitabine resistance	Fang et al. (2019)
		miR-221	Upregulated	Suppress PTEN expression in gemcitabine resistance	Richards et al. (2022)
		miR-181a			
		miR-21			
		miR-222			
		miR-92a			
	Non-small cell lung cancer	miR-103a-3p	Upregulated	Downregulates Bak1 to increase the cisplatin tolerance and inhibits cell death	Wang et al. (2021b)
					Zhang et al. (2021a)
	Breast cancer (ERα positivity)	miR-22	Upregulated	Causes resistance to tamoxifen	Gao et al. (2020)
	Colorectal cancer	miR-24-3p	Upregulated	Downregulates the CDX2/HEPH axis and hastens the cells' resistance to methotrexate	Zhang et al. (2021b)
		miR-181d-5p	Upregulated	Targets NCALD reducing the sensitivity to 5-FU	Wang et al. (2020)
		miR-625-3p	Upregulated	Blocks the CELF2/WWOX pathway	Zhang et al. (2022d)
	Ovarian cancer	miR-98-5p	Upregulated	Targets CDKN1A and increases cisplatin resistance	Guo et al. (2019)
	Gastric cancer	miR-522	Upregulated	Suppresses ALOX15 and decreases lipid-ROS accumulation	Zhang et al. (2020c)
	Colorectal cancer	miR-200b-3p	Downregulated	Enhances the sensitivity to 5-fluorouracil by targeting high mobility group protein 3	Yuan et al. (2022)
Radiotherapy resistance	Colorectal cancer	miR-93-5p	Upregulated	Stimulates nuclear accumulation of TGFβ by downregulating FOXA1 and reducing its promoter-binding interaction with TGFβ, consequently enhancing then proliferation and radiation-induced apoptosis	Chen et al. (2020b)
		miR-590-3p	Upregulated	Targets the PI3K/Akt signaling pathway, which is positively regulated by CLCA4	Chen et al. (2021b)
	Lung cancer	miR-196a-5p	Upregulated	Downregulates NFKBIA and promotes the malignant phenotype of radiation-resistant cells	Yao et al. (2023)
Immune regulation	Breast cancer	miR-92	Upregulated	Targets LATS2 and interacts with YAP1, attaches to the enhancer area of PD-L1 as nuclear translocation proceeds, and encourages resulting to T-cell death	Dou et al. (2020)
	Bladder cancer	Not clear	Not clear	Mediates the immune escape by regulating the expression of PD-L1/PD-1	Feng et al. (2023)
	Oral squamous cell carcinoma	miR-139-5p	Upregulated	Relates to immune cell infiltration	Wang et al. (2023)
	Prostate cancer	miR-320a	Upregulated	Regulates PTEN/PI3Kγ pathway to polarize the macrophages into M2 phenotype and accelerate the malignant behavior of cells	Zhao et al. (2022)

CAF-derived exosomes that affect tumor EMT are not only limited to miRNAs but also include related cytokines (Goulet et al., 2019; Al-Raimi et al., 2023), long-chain non-coding RNAs (Jin N. et al., 2020; Shi Z. et al., 2022; Zhang Y. et al., 2022), and proteins (Pan et al., 2022b; Liu X. et al., 2023). EMT is an important factor affecting tumor progression. Exploring its pathogenesis holds implications for the early diagnosis, treatment plans, and prognosis of patients with cancer. There is little research on the impact of CAF-derived exosomes on EMT, and further in-depth studies are needed in the future.

### 5.2 Role in tumor invasion and migration

As important indicators of the advancement of malignant tumors, invasion and migration have an impact on cancer prognosis and treatment. Wang et al. employed *in vitro* experiments and miRNA microarray analysis in an OS study to determine and validate the increase in miR-1228 levels in CAFs and their secreted exosomes, facilitating OS invasion and migration by downregulating endogenous SCAI mRNA and protein levels (Wang J. W. et al., 2019). Shi et al. found that miR-345-5p is a considerably elevated miRNA in exosomes derived from CAFs compared to exosomes obtained from normal fibroblasts. Through interaction with CDKN1A, exosomes mediate the transfer of miR-345-5p to CRC cells, promoting growth and metastasis (Shi et al., 2023). Simultaneously, CAF-derived exosomes are carriers of miR-21 that facilitate CRC transmission (Bhome et al., 2017). The ability to transfer highly expressed CAF-derived exosomal miRNAs to CRC cells, which then directly targets the 3′UTRs of the Runt-domain transcription factor 3 (RUNX3), was further demonstrated in an experiment. These exosomes also exhibited a higher miR-17-5p expression than exosomes derived from normal fibroblasts. Through its interaction with the MYC pro-oncogene and transforming growth factor-β1 (TGF-β1) at base pairs 1005–1296 promoter binding, RUNX3 promotes the progression of tumors by activating TGF-β signal pathways. Moreover, exosomal miR-17-5p is constantly released into CRC cells by autocrine TGF-β activating CAFs through the RUNX3/MYC/TGF-β1 signal, generating positive and negative feedback loops for CRC development (Zhang et al., 2020b). Oral squamous cell carcinoma (OSCC) is the most common malignant tumor in the head and neck region, with a high propensity for metastasis. Mauricio et al. in 2019 noted that extracellular vesicles formed from CAFs were crucial for encouraging OSCC cell migration and invasion (Dourado et al., 2019). In a subsequent investigation, Sun et al. found that OSCC overexpressed the CAF-derived exosomal miR-382-5p (Sun et al., 2019). According to a recent study, miR-146b-5p is also overexpressed in OSCC. Subsequent luciferase assay tests confirmed that it can specifically target the 3′UTR of HIPK3, leading to the suppression of HIPK3 and its involvement in the migration and invasion process of OSCC cells. (He et al., 2023). miRNAs in the DLK1-DIO3 imprinting area, including miR-329-3p, miR-380-3p, miR-410-5p, and miR-431-5p, were found to be upregulated in hepatocellular carcinoma (HCC) cells co-cultured with exosomes produced from CAFs. These miRNAs inhibited HHIP expression, weakened cell adhesion, and enabled cell migration and invasion (Jin et al., 2022). In HCC, miR-20a-5p has been demonstrated to have a comparable impact (Qi et al., 2022). Other studies about CAF-derived exosomal miRNAs’ role in invasion and migration are shown in Table 1 (Xu et al., 2019; Yang F. et al., 2020; Guo et al., 2020; Shi et al., 2020; Liu et al., 2021; Zhou et al., 2022; Wu et al., 2023).

Tumor angiogenesis is an important form of nutrient acquisition and metastasis in tumor cells (Bhat et al., 2021). Researchers have found that exosomal miRNAs derived from CAFs can also participate in the process of promoting tumor angiogenesis. For example, CAF-derived exosomes promote angiogenesis by delivering miR-21 to endothelial cells in multiple myeloma (Miaomiao et al., 2023). miR-135b-5p is upregulated, promoting angiogenesis in CRC cells by inhibiting thioredoxin-interacting proteins (Yin et al., 2021). Similarly, miR-135b-5p downregulates FOXO1 in colorectal adenocarcinoma and encourages the migration and proliferation of human umbilical vein endothelial cells, resulting in angiogenesis (Dai et al., 2022). Lymphatic metastasis is another important method of tumor metastasis besides hematogenous metastasis. The reduction of CAF-derived exosomal miR-100-5p in esophageal squamous cell carcinoma (ESCC) may promote the growth, migration, and invasion of tumor-related lymphatic endothelial cells by increasing the expression of IGF1R/PI3K/AKT, ultimately leading to the development of lymphatic vessels (Chen et al., 2023) (Figure 2).

### 5.3 Role in tumor growth

As the primary constituents of the cancer matrix, CAFs can secrete exosomes to affect the processes relevant to tumor growth regulation (Figure 2). The regulatory process of tumor growth includes aspects such as cell proliferation, reprogramming of cell metabolism, and obtaining the stem cell phenotype.

The exosomal miRNAs generated from CAFs influence the growth of tumor cells. In non-small cell lung cancer, exosomes derived from the CAFs express miR-20a more frequently, which serves as a conduit to infiltrate tumor cells. Targeting PTEN increases the PI3K/AKT pathway’s activity, promoting proliferation (Shi L. et al., 2022). Cui et al. conducted a study where they incubated ESCC cell lines (TE-1 and KYSE-150) with exosomes formed from CAFs. The results indicated that increased RIG-I/IFN-β expression could increase cell proliferation. Additionally, they observed that exosomes derived from CAFs prevented cell apoptosis (Cui et al., 2022). A previous study suggested that miRNAs could be crucial to cellular apoptosis, warranting further investigation in subsequent studies (Jin et al., 2021). In breast cancer, miR-500a-5p can migrate from CAFs to cancer cells via exosomes. Once inside the cancer cells, it attaches itself to USP28, a ubiquitin-specific peptidase, to promote cell division (Chen B. et al., 2021). It was discovered in CRC that miR-135b-5p stimulated tumor cell proliferation and angiogenesis (Yin et al., 2021). Furthermore, miR-181b-3p is involved in colorectal tumorigenesis (Jiang Y. et al., 2023).

The metabolism of tumor cells exhibits significant departures from that of healthy cells, a deviation that underlies the emergence of a wide array of malignant tumor symptoms (Lv et al., 2021; Zanotelli et al., 2021; Pavlova et al., 2022). In a study by Zhao et al., exosomes derived from prostate cancer (PCa) CAFs had higher levels of miR-22 and miR-125b, which decreased mitochondrial oxidative phosphorylation and caused changes akin to hypoxia in the TME that can result in metabolic alterations in cancer cells (Zhao et al., 2016).

Tumor cells have strong plasticity (Chiodi and Mondello, 2020). The generation of stem cell-like characteristics is an important driving factor for tumor cells to self-renew, have clonal tumor initiation ability, and possess long-term proliferation potential (Han et al., 2020; Huang et al., 2020; Suva and Tirosh, 2020; Kumar et al., 2022). In exosomes secreted by CAFs, miR-34c-5p expression was significantly reduced in laryngeal carcinoma. *In vivo* and *in vitro* research has shown that miR-34c-5p regulates the stem cell-like characteristics of laryngeal cancer cells (Wang et al., 2022c). When the Wnt/β-catenin pathway was activated in CRC, the expression of CAF-derived exosomal miR-92a-3p rose and directly suppressed FBXW7 and MOAP1 expression to prevent mitochondrial apoptosis, causing CRC cells to produce stem cell characteristics (Hu et al., 2019). This can significantly impact the cancerous characteristics of tumors, including their ability to proliferate, invade, alter their shape and form plate colonies, become tumorigenic, and express high levels of tumor stem cell genes that are important for tumor development (Machida, 2020).

### 5.4 Role in therapy resistance

Although there have been notable advancements in anti-cancer treatment, drug resistance associated with molecular and clinical recurrence remains prevalent. As a result, many patients resort to different treatment approaches, yet their prognosis remains unfavorable. Innate and/or acquired resistance mechanisms severely restrict the clinical efficacy of anti-cancer therapy (Hofmann et al., 2023). Thus, it is imperative to investigate the precise mechanisms of treatment resistance in clinical practice to create significant changes in tumor treatment modalities. Understanding these pathways may aid in forecasting the development of clinical drug resistance and identifying alternate therapeutic approaches. Exosomal miRNAs derived from CAFs have been implicated in tumor therapy resistance (Figure 2), providing valuable insights into resistance mechanisms from the TME perspective.

Exosomal miRNAs produced from CAFs may contribute to developing chemotherapy resistance in tumors. Gemcitabine (GEM) is frequently used to treat PC (Motoi et al., 2019; Beutel and Halbrook, 2023; Jiang X. et al., 2023). However, individuals with PC still face the difficulty of developing resistance to GEM (Liang et al., 2017). According to a study by Fang et al. on the role of exosomal miRNAs from CAFs in regulating drug resistance, CAFs are intrinsically resistant to GEM. Subsequent GEM therapy demonstrated elevated miR-106b levels in CAFs and CAF exosomes directly targeting TP53INP1, resulting in GEM resistance in cancer cells (Fang et al., 2019). Richards et al. identified five different forms of short RNAs in another experiment: miR-221, miR-181a, miR-21, miR-222, and miR-92a. During GEM therapy, these miRNAs considerably increased the production of CAF-derived exosomes targeting PTEN. *In vitro* investigations demonstrated that these CAF exosomes might suppress PTEN expression (Richards et al., 2022). The experiment found that CAFs derived from non-small cell lung cancer (NSCLC) exhibit inherent resistance to cisplatin treatment (Kryczka et al., 2021; Shi L. et al., 2022). CAF-derived exosomes play a role in developing chemotherapy resistance in NSCLC cells by moving from CAFs to NSCLC cells. However, this chemotherapy resistance can be reversed by suppressing the miR-103a-3p gene. Subsequent studies have shown that miR-103a-3p directly targets Bak1. Downregulating Bak1 increases cisplatin tolerance in NSCLC cells and inhibits cell death. Furthermore, miR-103a-3p can be packaged into exosomes by the RNA-binding protein PUM2 (Zhang T. et al., 2021; Wang H. et al., 2021). For breast cancer with estrogen receptor alpha (ERα) positivity, tamoxifen is still the most effective treatment. However, a significant proportion of patients still face metastases and recurrence, along with resistance to tamoxifen, which poses significant therapeutic challenges. In a previous study, Gao et al. found that CD63 + CAFs caused patients with breast cancer to become resistant to tamoxifen by secreting miR-22. This suggested that CD63 + CAFs could be a potential target for therapy to improve tamoxifen sensitivity (Gao et al., 2020). Further details about the role of therapy resistance are provided in Table 1 (Guo et al., 2019; Zhang H. et al., 2020; Gao et al., 2020; Zhang H. W. et al., 2021).

Radiotherapy, an important tumor treatment method, has demonstrated therapeutic effects influenced by miRNAs in CAF-derived exosomes. High miR-93-5p CAF-derived exosomes stimulate the nuclear accumulation of TGFβ by downregulating FOXA1 and reducing its promoter-binding interaction with TGFβ, consequently enhancing the proliferation and shielding of CRC SW480 cells from radiation-induced apoptosis (Chen X. et al., 2020). Another study found that miR-590-3p can boost the radiation resistance of CRC by targeting the PI3K/Akt signaling pathway, which is positively regulated by CLCA4 (Chen X. et al., 2021). In lung cancer, CAF-derived exosomal miR-196a-5p downregulates NFKBIA, promoting the malignant phenotype of radiation-resistant cells and contributing to radiation resistance (Yao et al., 2023).

Studying the mechanism of tumor therapy resistance mediated by CAF-derived exosomal miRNAs can provide new targets for improving the sensitivity of tumor cells to radiotherapy and chemotherapy. However, current studies mainly focus on resistance to radiotherapy and chemotherapy, while studies on biological targeted therapy, immunotherapy, and resistance caused by tumor cell heterogeneity have not yet emerged. Thus, these may be good research directions, necessitating further exploration in the future.

### 5.5 Role in immune regulation

Cancer is a complex ecosystem in which the interaction between cancer and host cells can affect the disease progression and treatment response. Besides cancer cells, immune cells are arguably the most complex players in solid tumors, and their activity can range from anti-tumorigenic to tumorigenic (Dumauthioz et al., 2018; Oliveira and Wu, 2023). Cancer cells employ diverse strategies to thwart immunological defenses as their tumors grow, including downregulating the antigen presentation pathway and triggering the production of immune checkpoint molecules that suppress immune responses (Jhunjhunwala et al., 2021). In addition, cancer cells exploit immune cells such as neutrophils, macrophages, and regulatory T cells to create an immunosuppressive TME (Lei et al., 2020). This, in turn, promotes immune escape, remodels the ECM, and supports cancer progression and treatment resistance. Therefore, abnormal immune responses are considered as markers for cancer (Khalaf et al., 2021; Mafi et al., 2021; Peng et al., 2022). Recent research has also demonstrated that miRNAs in exosomes produced from CAFs regulate the tumor immune system and offer therapeutic targets for cancer.

A recent experiment discovered a novel mechanism for inducing immunosuppression in the TME. Increased expression of miR-92 and greater levels of programmed cell death receptor ligand 1 (PD-L1) were detected in breast cancer cells treated with CAF-derived exosomes produced from human breast cancer cells (Feng et al., 2021). LATS2 is the miR-92 target gene, and immunoprecipitation has demonstrated the interaction between LATS2 and YAP1 (Jin D. et al., 2020). Tumor transcriptional activity was increased when YAP1 was attached to the enhancer area of PD-L1 as nuclear translocation proceeded. To a certain extent, this effect hampered the proliferation and strongly encouraged T-cell death (Dou et al., 2020). In bladder cancer, CAF-derived exosomes have also been confirmed to mediate the immune evasion of cancer cells by modulating PD-L1/PD-1 expression (Feng et al., 2023). However, the specific molecules that play a role in the exosomes still need to be further explored, and the participation of miRNAs is a potential research direction. The immune regulatory pathway may be a special mechanism by which CAFs promote OSCC proliferation. Wang et al. conducted bioinformatics analysis on foreign sequencing data from CAF sources and identified all the candidate genes related to tumor immunity. Hsa-miR-139-5p is one of the target genes, and its expression differences and prognostic significance are well-established in the TCGA dataset. The study found that has-miR-139-5p was related to immune cell infiltration, and its low expression promotes the development of OSCC, which is closely related to patient survival (Wang et al., 2023). M2-type macrophages are activated by cytokines such as IL-4 and IL-13. They have an enhanced ability to engulf particles due to the secretion of anti-inflammatory cytokines, including TGF-β and IL-10. These cytokines promote the generation of Th2 cells and contribute to immune regulation and angiogenesis processes. Consequently, they facilitate the rapid dissemination of tumors (Chen D. et al., 2021; Christofides et al., 2022). CAF-derived exosomal miR-320a has been shown to regulate the PTEN/PI3Kγ pathway to polarize the macrophages into M2 phenotype, promoting the malignant behavior of PCa cells (Zhao et al., 2022) (Figure 2). Tumor immune regulation is crucial for studying the mechanisms of tumor occurrence in the body, and it is of great significance in the development of tumor treatment methods, the generation of treatment resistance, and patient prognosis. CAF-derived exosomal miRNAs have shown excellent research value in this field. With the deepening of various studies, new targets for tumor treatment are likely to emerge.

## 6 Tumor inhibitory effect of CAF-derived exosomal miRNAs

Extensive research has demonstrated the considerable tumor-suppressive effects of several CAF-derived exosomal miRNAs across tumor types.

Androgen deprivation therapy (ADT) is the cornerstone treatment for advanced PCa (Shafi et al., 2013). Despite the initial good response, castration resistance and metastatic progression inevitably occur (Li Q. et al., 2021). Through the EGFR/ERK pathway, exosomal miR-146a-5p produced from CAFs can prevent PCa cells from metastasizing when treated with ADT (Zhang et al., 2020d). In OSCC, exosomes derived from CAFs exhibited reduced expression of miR-34a-5p. In xenograft trials, OSCC cell carcinogenesis can be prevented by overexpressing miR-34a-5p in CAFs. It was also demonstrated that miR-34a-5p could bind to its direct downstream target AXL, and prevent the proliferation and metastasis of OSCC cells (Li et al., 2018). Peritoneal metastases are frequent and an extensive hallmark of ovarian cancer (OC) (Pascual-Anton et al., 2021). Overexpression of miR-29c-3p in exosomes produced from CAF suppresses tumor metastasis by amplifying its impact on the direct target, matrix metalloproteinase 2 (MMP2) (Han et al., 2023). Studies on breast cancer have shown that the concentrated presence of miR-16 and miR-148a in exosomes derived from CAFs has beneficial effects on anti-tumor cell activity and anti-metastasis (Wu et al., 2020). Similarly, miR-4516 targeted FOSL1’s tumor-promoting activity to inhibit triple-negative breast cancer (Kim et al., 2020). CAF-derived exosomal miRNA also plays a certain role in enhancing drug sensitivity. For example, miR-200b-3p was upregulated in exosomes derived from hypoxic CAFs, improving the sensitivity of CRC to 5-fluorouracil by targeting high mobility group protein 3 (Yuan et al., 2022). In addition, miR-195 in CAF-derived exosomes improved the survival rate in rat models of intrahepatic cholangiocarcinoma (Li et al., 2017). The inhibition of miR-320a via the HIF1 α/VEGFA axis on endometrial cancer has also been verified (Zhang N. et al., 2020). miR-3188 has also been proven to engage in the proliferation of head and neck cancer cells (Wang X. et al., 2019), and miR-1-3p has been shown to inhibit migration and invasion of breast cancer cells (Tao et al., 2021).

The multifaceted roles of miRNA in cancer development and its treatment vary significantly among different types of tumors. Therefore, it is essential to understand the specific types and mechanisms of miRNA to effectively diagnose and treat tumors (Table 1).

## 7 The potential of CAF-derived exosomal miRNAs in diagnosis and treatment

CAF-derived exosomal miRNAs have corresponding roles in tumor cell growth, migration and invasion, EMT, immune regulation, and treatment. These findings indicate their rich potential in tumor early diagnosis, treatment, and prognosis prediction.

Regarding diagnosis, the distinct composition of miRNAs in exosomes from tumor and normal tissues allows for identifying biomarkers. This is possible because exosomes are found in various bodily fluids. Diagnostic criteria can be established by analyzing the miRNAs that are significantly altered in the specific tumors. The patient’s discomfort can be reduced using non-invasive technology to collect bodily fluids, separate exosomes for miRNA identification, and determine the type of tumor. Moreover, they can serve as an early detection method in scenarios where pathology and imaging fail to yield valuable insights regarding microtumors (Gerloff et al., 2022; Takizawa et al., 2022). Hiroshi and his colleagues demonstrated that matrix miR-21 is more crucial for the progression of GC than tumor cell miR-21 (Uozaki et al., 2014). Additionally, CAF-derived miRNAs help with clinical tumor staging to create more individualized diagnoses and treatment regimens. A study has demonstrated that miR-92a-3p is highly expressed in CAF-derived exosomes in liver cancer patients with a diameter greater than 5 cm, and it is more expressed in patients with BCLC B/c phase (Liu X. et al., 2022). A strong theoretical foundation for liquid biopsy can also be provided by investigating CAF-derived exosome miRNAs. Exosomes can potentially be therapeutic agents due to their superior biocompatibility and circulatory stability.

Exosome-derived miRNAs have attracted a lot of interest in tumor treatment studies due to the specificity of their structure and mode of action. However, recent research primarily focused on the exosomal miRNAs from tumor cells (Jabbari et al., 2020; Kok and Yu, 2020; Huang et al., 2022; Wan et al., 2022), with relatively little investigation into the origins of CAFs. Due to their direct effects on genetic material, it is evident that CAF-generated exosomal miRNAs have significant potential in tumor treatment, as indicated in this paper. Given their robust structure, exosomes can efficiently transport processed miRNAs as nanocarriers, decreasing the likelihood of degradation (Zhang et al., 2022e). This characteristic makes CAF-derived exosomes crucial for biological information transfer within TME (Peng et al., 2021). Following absorption by adjacent receptor cells, these receptor cells undergo a sequence of events, including controlling signal pathways and targeting particular genes. This method substantially impacts drug resistance, immunological response, metastasis, and tumor cell proliferation.

Based on the current evidence, exploring CAF-derived exosomes holds promise for the development of innovative methods for tumor diagnosis and therapy.

## 8 Discussion

Previous studies on cancers have primarily focused on tumor cells. Recently, researchers started to investigate the relationship between different components of TME and malignancies, in addition to cancer cells. CAF-derived exosomes, a key TME mediator, are essential for developing and spreading cancer and can be considered as a channel for information exchange within TME. Due to their influence on gene transcription and expression control, miRNAs—which are extensively found in body fluids and tissues—have also drawn much attention related to tumor growth studies (Gjorgjieva et al., 2019). In the last 5 years, there has been a notable shift in the emphasis of research concerning the effects of exosomal miRNAs derived from CAFs on tumors. Presently, a multitude of studies are in the advanced stages of development. It is known that exosomal miRNAs produced from CAFs have equivalent functions in the development, invasion, and migration of tumor cells apart from EMT, immunological control, and therapy. These imply that the miRNAs have rich development prospects in early tumor diagnosis, treatment, and prognosis prediction.

Nevertheless, research on the application of CAF-derived exosomes as biomarkers in diagnosis and treatment still faces some challenges. First, no established extraction method reliably produces exosomes with high levels of purity when it comes to exosome separation and purification techniques. Moreover, the integrity processing of exosomes obtained from the existing processes is not well-quantified, which may greatly affect their accuracy and efficiency in diagnosis. Second, the complex characteristics of TME require further investigation to determine the exact mechanism through which miRNAs obtained from CAF-derived exosomes interact with tumor cells, thereby influencing the behavior of cancerous tumors. In addition, more investigations are required to ascertain the tumor-specificity of miRNAs in CAF-derived exosomes extracted from various sources. Third, there is a lack of clear experimental evidence regarding the capacity of exosomes to consistently remain present in bodily fluids and generate potent therapeutic effects. Thus, CAF-derived exosomal miRNAs remain viable and efficient targets for cancer therapy due to their distinct function in malignancies.

## 9 Conclusion

This study focused on elucidating the functions of miRNAs produced from CAFs in promoting malignant features in tumors, including EMT, migration and invasion, tumor growth, treatment resistance, and immunomodulation. Additionally, we emphasized the inhibitory effect of CAF-derived exosomal miRNAs.

We emphasize that with comprehensive and creative research, the functions of CAF-derived exosomal miRNAs in tumor progression can be further clarified, providing a robust theoretical framework for clinical diagnosis and treatment applications. This will relieve patients' intense psychological and physical suffering, in addition to the substantial financial strain that malignant tumors impose.

## Figures and Tables

**Table:** 

**CAF**	cancer-associated fibroblasts
**TME**	tumor microenvironment
**ECM**	extracellular matrix
**EMT**	epithelial-mesenchymal transition
**JAMA**	Journal of the American Medical Association
**NK**	cells, natural killer cells
**EVs**	extracellular vesicles
**FAP**	fibroblast activating protein
**α-SMA**	α-smooth muscle actin
**CSCs**	cancer stem cells
**PDPN**	podoplanin
**FAP-α**	fibroblast activating protein
**PDGFR α or β**	platelet-derived growth factor receptor α or β
**α- SMA**	α- smooth muscle actin
**FSP-1**	fibroblast specific protein-1
**VIM**	vimentin
**COL1A1/COL1A2**	collagen 1a1/1a2
**LUM**	lumican
**DCN**	decorin
**RNA pol II**	RNA polymerase II
**AGO2**	argonaute protein
**RISC**	RNA-induced silencing complex
**E/M**	mixed epithelial and mesenchymal
**CRC**	colorectal cancer
**DLK1-DIO3**	delta-like 1 homologous deiodinase, iodothyronine 3
**RUNX3**	Runt-domain transcription factor 3
**TGF-β1**	transforming growth factor β1
**OSCC**	oral squamous cell carcinoma
**HCC**	hepatocellular carcinoma
**ESCC**	esophageal squamous cell carcinoma
**USP28**	ubiquitin-specific peptidase 28
**PCa**	prostate cancer
**GEM**	gemcitabine
**NSCLC**	non-small cell lung cancer
**ERα**	estrogen receptor alpha
**PD-L1**	programmed cell death receptor ligand 1
**PD-1**	programmed cell death protein 1
**ADT**	Androgen deprivation therapy
**OC**	ovarian cancer
**MMP2**	matrix metalloproteinase 2
